# A Computational Model of a Microfluidic Device to Measure the Dynamics of Oxygen-Dependent ATP Release from Erythrocytes

**DOI:** 10.1371/journal.pone.0081537

**Published:** 2013-11-27

**Authors:** Richard J. Sove, Nour Ghonaim, Daniel Goldman, Christopher Gerald Ellis

**Affiliations:** 1 Department of Medical Biophysics, University of Western Ontario, London, Ontario, Canada; 2 Biomedical Engineering Graduate Program, University of Western Ontario, London, Ontario, Canada; University of Arizona, United States of America

## Abstract

Erythrocytes are proposed to be involved in blood flow regulation through both shear- and oxygen-dependent mechanisms for the release of adenosine triphosphate (ATP), a potent vasodilator. In a recent study, the dynamics of shear-dependent ATP release from erythrocytes was measured using a microfluidic device with a constriction in the channel to increase shear stress. The brief period of increased shear stress resulted in ATP release within 25 to 75 milliseconds downstream of the constriction. The long-term goal of our research is to apply a similar approach to determine the dynamics of oxygen-dependent ATP release. In the place of the constriction, an oxygen permeable membrane would be used to decrease the hemoglobin oxygen saturation of erythrocytes flowing through the channel. This paper describes the first stage in achieving that goal, the development of a computational model of the proposed experimental system to determine the feasibility of altering oxygen saturation rapidly enough to measure ATP release dynamics. The computational model was constructed based on hemodynamics, molecular transport of oxygen and ATP, kinetics of luciferin/luciferase reaction for reporting ATP concentrations, light absorption by hemoglobin, and sensor characteristics. A linear model of oxygen saturation-dependent ATP release with variable time delay was used in this study. The computational results demonstrate that a microfluidic device with a 100 µm deep channel will cause a rapid decrease in oxygen saturation over the oxygen permeable membrane that yields a measurable light intensity profile for a change in rate of ATP release from erythrocytes on a timescale as short as 25 milliseconds. The simulation also demonstrates that the complex dynamics of ATP release from erythrocytes combined with the consumption by luciferin/luciferase in a flowing system results in light intensity values that do not simply correlate with ATP concentrations. A computational model is required for proper interpretation of experimental data.

## Introduction

In the human body, the regulation of oxygen transport is an important process to ensure that the demands for oxygen are met. The circulatory system has various mechanisms responsible for the delivery of oxygen to regions of high metabolic activity. Oxygen regulation can occur on a large scale or locally within specific tissue. The vessels responsible for local regulation mechanisms are known to be the small arterioles and capillaries, which comprise the microcirculation.

Erythrocytes have been shown to release ATP in response both to low erythrocyte hemoglobin oxygen saturation (SO_2_) [1], [2] and to increased shear stress on the erythrocyte membrane [1], [3]. Both mechanisms are suspected to be involved in the regulation of flow in the microcirculation. In a recent study, the dynamics of shear-dependent release of ATP from erythrocytes was measured by flowing erythrocytes through a constriction in a microfluidic device to induce a brief period of increased shear stress [3]. The authors report that the ATP release occurred within 25 to 75 milliseconds after the period of increased shear.

The oxygen-dependent release of ATP is hypothesized to be a mechanism involved in regulating the distribution of oxygen within the microvasculature, where the erythrocyte plays the role of the oxygen sensor [4]. An important aspect of this hypothesis is the time required for ATP release to occur following a change in SO_2_, since this determines the spatial accuracy with which the erythrocyte can signal for vasodilation. Our ultimate goal is to measure the dynamics of oxygen-dependent release of ATP by applying a similar approach to the study by Wan et al. [3]. In the place of the constriction in the microfluidic device, we will use an oxygen permeable membrane to cause a rapid change in SO_2_ as the erythrocytes flow through the channel.

Understanding this mechanism may also have important clinical implications. In patients with type II diabetes, ATP release is known to be significantly lower for the same change in oxygen saturation [5]. However, we do not know if the time course is also altered in type II diabetes and other cardiovascular diseases [5].

Several concerns must be assessed to design an effective microfluidic device for the study of oxygen-dependent ATP release. First, we must determine whether a practical device can cause a sufficient drop in SO_2_ to cause ATP release and whether the change in SO_2_ is fast enough to measure the dynamics. Second, we must assess whether the experimental setup can resolve ATP release times as fast as 25 milliseconds. In this study, we will describe a computational model of a microfluidic system to address the aforementioned concerns.

**Figure 1:**
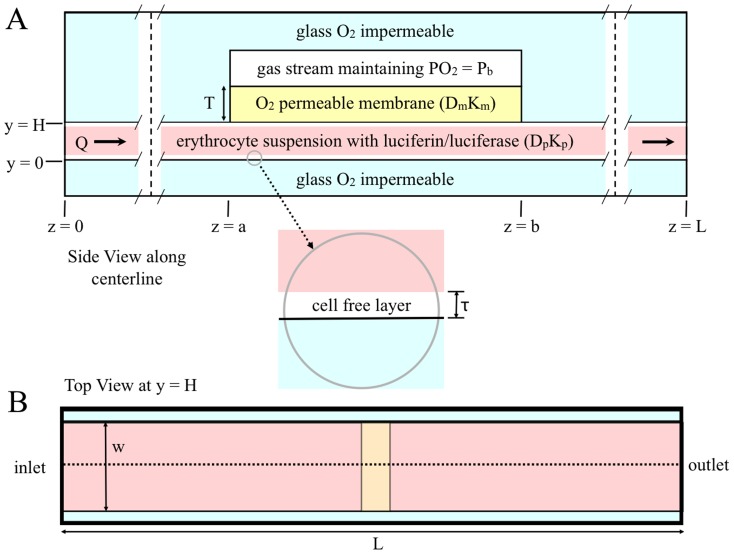
Two-dimensional representation of a microfluidic device for measuring oxygen-dependent ATP release from erythrocytes. A. shows the side view along the centerline. This view of the diagram is to scale; the vertical dashed lines indicate where the diagram is broken to allow for the entire device to be shown. B. shows the top view; the dotted line indicates the centerline. Note: this view of the diagram is not to scale.

The microfluidic device should be designed such that the majority of the channel is oxygen impermeable and should contain an oxygen permeable region at which the oxygen partial pressure (PO_2_) is held at a lower PO_2_ than that of the blood. As the blood passes the oxygen permeable region, oxygen diffuses out of the blood causing a drop in SO_2_. We implemented a similar approach to decrease PO_2_ in a 2011 study of methods for localized oxygen delivery *in vivo* [6]. In the current study, oxygenated blood would flow through the channel at a constant flow rate to produce a steady state distribution of SO_2_ and ATP levels within the channel. A possible design for a microfluidic device is shown in Fig. 1.

To measure ATP levels, the blood in the system will be mixed with a solution of luciferin and luciferase as it enters the microfluidic device. Luciferin and luciferase react with ATP, producing light proportional to the concentration of ATP [7], [8]. The light intensity levels will be measured using a microscope and still-framed camera with appropriate exposure time. Since ATP release will be measured in steady state, changes in time can be measured as changes in position along the channel. Exploiting time in terms of distance allows for the measurement of time on the order of milliseconds without having to use a light detection system with high temporal resolution.

In this paper we present a mathematical model of the microfluidic system to aid in the design the experimental setup and to gain further insights into this phenomenon. The mathematical model will also give us the tools necessary to analyze future experimental results.

## Materials and Methods

### Overview of Model

The model is comprised of a number of coupled modules, each corresponding to a component of the mass transport and light detection system. The first underlying module in this simulation is a model for the hemodynamics of system. This module determines how the blood plasma and erythrocytes will behave and interact as they flow through the microfluidic device. Based on the hemodynamic module, a second module for oxygen transport was developed. This module describes the convection and diffusion of oxygen within the blood and it also includes the relationship between PO_2_ and SO_2_.

A module for ATP release was added based on both SO_2_ and the hemodynamic module. The ATP module describes the rate of ATP release throughout the microfluidic device. A simple linear relationship between SO_2_ and ATP release rate was used, as this is sufficient to determine whether the device is capable of measuring ATP. A linear model was also used in a previous study [9]. A time delay term was included in this module, which accounts for the time required for ATP to be released following the change in SO_2_. This term was included to ensure the device was capable of measuring the dynamics of ATP release. A further module for ATP transport was constructed based on the diffusion and convection of ATP in the blood plasma. The degradation of ATP due to the reaction with the luciferin and luciferase solution was also included.

In addition to the degradation of ATP, a module for the light generated by the luciferin and luciferase reaction was developed. This luminescence module was based on the kinetics of the reaction between ATP and the luciferin and luciferase solution. From this module, the amount of light coming from the system was determined and used to calculate the light intensity signal that would be measured by a digital camera. This module includes both the attenuation of light as it passes through hemoglobin based on extinction coefficients and the efficiency of a scientific digital camera for measuring light levels at a wavelength of 560 nm. In this configuration, image acquisition is taken from the top of the channel to acquire the information closest to the oxygen permeable membrane. A detailed mathematical description of each module follows below.

### Hemodynamic Module

The geometry of this model is presented in Fig. 1; it considers a slice in the yz-plane along the centerline. Blood flow is assumed to be viscous and in steady state. The module incorporates a core region of blood flow mixed with erythrocytes where hematocrit is assumed to be constant and a cell-free plasma layer along the channel walls where hematocrit is assumed to be zero.

In this model, velocities in the x and y directions are assumed to be zero. The velocity profile of the blood plasma was derived from the Navier-Stokes equations for fluid dynamics. Steady state is assumed, and the shear stress between the cell-free layer and core region is assumed to be equal at the interface. Equation 1 governs the flow velocity of the blood plasma.
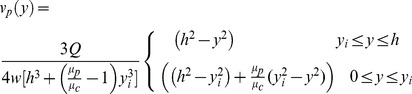
(1)


In this equation Q is the flow rate, h is half the height of the channel, w is the width of the channel, y is the distance from the center of the channel, y_i_ is the position of the interface between the cell-free layer and the cell-plasma mixture, μ_p_ is the dynamics viscosity of the cell-free layer and μ_c_ is the dynamic viscosity of the cell-plasma mixture and is calculated empirically based on the work of Pries et al [10].

The erythrocytes flow at a velocity slower than the plasma due to slipping. The slip velocity, slp, defines the magnitude of slipping between the plasma and erythrocytes [11]. Equation 2 governs the flow velocity of the erythrocytes. 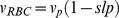
(2)


Tube hematocrit in a flowing system is different from inlet or discharge hematocrit, and is defined as the fraction of erythrocytes flowing in the core region of blood flow [12]. Equation 3 defines the relationship between tube hematocrit (H_T_), discharge hematocrit (H_D_) and the channel height. τ is the cell-free plasma layer thickness.
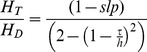
(3)


### Oxygen Transport Module

Oxygen movement is dictated by diffusion and convection in a flowing system. In this module, oxygen is assumed to diffuse in the y-direction and to move by convection in the z-direction. Oxygen is also transported by erythrocytes through the binding of oxygen to hemoglobin. Equation 4a governs oxygen transport in the cell-free plasma layer and Equation 4b governs oxygen transport in the blood mixture [11]. 
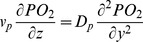
(4a)

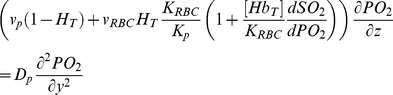
(4b)


PO_2_ is the partial pressure of oxygen; D_p_ is the diffusion coefficient of oxygen in blood plasma; K_p_ and K_RBC_ are the oxygen solubility in the cell-free plasma layer and blood mixture respectively; [Hb_T_] is the total hemoglobin concentration, and 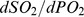 is the derivative of the Hill equation (see Eq. 5 below). The initial condition is described by Equation 4c and the boundary conditions are described by Equation 4d. The oxygen permeable membrane is present on the upper wall of the channel and extend from z  =  a to z  =  b. D_m_ and K_m_ are the diffusion coefficient and solubility of the oxygen permeable membrane respectively. T is the thickness of the oxygen permeable membrane. 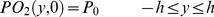
(4c)

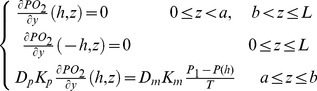
(4d)


SO_2_ was calculated based on the Hill equation, and depends on the PO_2_ output of the oxygen transport module. Equation 5 is the Hill equation, where N is the Hill coefficient, which characterizes the binding cooperativity of hemoglobin, and P_50_ is the PO_2_ at 50% saturation. 
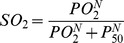
(5)


### ATP Release Module

This module describes ATP release rates and assumes that the relationship between ATP release rate and SO_2_ is linear with a minimum release rate (R_min_) when hemoglobin is fully saturated and a maximum release rate (R_max_) when it is fully de-saturated. Finally, the module assumes that there is a delay between the change in saturation and the change in ATP release rate. Equation 6 governs ATP release rate for these assumptions, where t_d_ is the time delay. 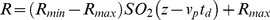
(6)


### ATP Transport Module

This module assumes that ATP diffuses in the y-direction and moves by convection in the z-direction. It assumes that ATP cannot diffuse through the erythrocyte membrane. This module includes erythrocytes as an ATP source and the depletion of ATP due to the reaction with luciferin and luciferase. Equation 7 governs ATP transport, where [ATP] is the concentration of ATP; D_ATP_ is the diffusion coefficient of ATP in blood plasma and k_t_ is the reaction rate constant for the luciferin and luciferase reaction with ATP. 
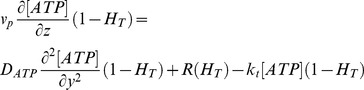
(7)


The differential equation was solved assuming that there is no ATP flux at the channel walls and that the luciferin and luciferase solution is being mixed at the inlet of the channel. It is assumed that there is no ATP entering the channel at the inlet and there is no ATP flux at the O_2_ permeable membrane.

### Luminescence Module

The luminescence in this system is derived from the kinetics of the reaction between ATP and luciferin/luciferase. Equation 8 shows the complete reaction [8]. 
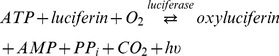
(8)


This reaction has first order kinetics such that the rate of reaction only depends on the concentration of ATP [7]; therefore, the rate at which light is produced is proportional to the rate of ATP consumption. For the amount of ATP released from erythrocytes, the concentration of the luciferin and luciferase solution will be much great than that of ATP; therefore product inhibition can be assumed to be negligible. Equation 9 describes the rate at which light is produced, where α is the quantum efficiency, V is the volume of each voxel, N_A_ is Avogadro's number and k_t_ is the rate constant of the reaction [8]. The reaction constant k_t_ varies typically between 0.1–1 s^−1^ [8]. 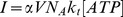
(9)


### Optics Module

Photon detection is based on the number of photons that reach the digital camera's charge-coupled device (CCD) and the device's ability to undergo the photoelectric effect. This module accounts for photon attenuation by hemoglobin and the fact that a point source emits light in all directions. Equation 10 describes the number of photons acquired per pixel; where t_s_ is the camera's shutter speed; A_pixel_ is the surface area of the pixel and κ is the attenuation coefficient. 
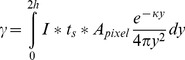
(10)


It is known that oxyhemoglobin and deoxyhemoglobin have different attenuation coefficients depending on wavelength. Equation 11 describes the light attenuation coefficient through the blood mixture as a function of saturation, where κ_p_, κ_Hb_ and κ_O2Hb_ are the attenuation coefficients of the blood plasma, deoxyhemoglobin and oxyhemoglobin respectively at the wavelength of light produced by the luciferin/luciferase reaction with ATP, 560 nm. 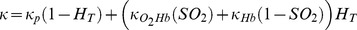
(11)


The signal read by the device comes from the amount of electrons released by the photons collected from the photoactive region of each well of the CCD. Thus the signal depends on the efficiency of the photoelectric effect (η) and the amount of photons collected on the surface of each well. Equation 12 describes the signal read by the device, where S_total_ is measured in electrons.
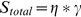
(12)


The device reads out each well and converts the number of electrons measured into a digital signal that depends on the memory information of the device. Equation 13 gives a measurement of the output signal relative to the maximum measurable signal by the CCD; this will be referred to as the relative output signal. The full-well capacity (FWC) of the CCD is the maximum number of electrons that each well can hold. 
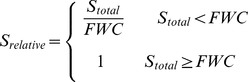
(13)


### Simulations

All numerical analyses were done in Mathworks MATLAB 7.11.0 (R2010b). Grid spacing for the model was 321×4000 (y-z). The parameters of the model are specified in Table 1. All simulations reached a stable solution. The default simulation was performed with a grid spacing of 321×4000, 642×8000 and 1284×16000; these simulations reached the same stable solution.

**Table 1:** Model parameters used in the simulations.

Parameter	Variable	Value
Flow Rate (µL/min)	Q	7.8
Device Height (µm)	H	100
Device Width (µm)	w	1500
Plasma Viscosity [12] (Pa.s)	μ_p_	0.001
Plasma Layer Position (µm)	y_i_	49
Slip Coefficient [11]	slp	0.1
Discharge Hematocrit	H_D_	0.2
Plasma Layer Thickness (µm)	τ	1
O_2_ Diffusivity in Plasma [11] (µm^2^/s)	D_p_	2750
O_2_ Solubility in RBCs [11] (µM/mmHg)	K_RBC_	1.47
O_2_ Solubility in Plasma [11] (µM/mmHg)	K_p_	1.33
Total Heme Concentration [11] (µM)	[Hb_T_]	5350
Inlet PO_2_ (mmHg)	P_0_	150
O_2_ Permeable Membrane PO_2_ (mmHg)	P_b_	10
O_2_ Permeable Membrane Start Position (µm)	a	7000
O_2_ Permeable Membrane End Position (µm)	b	7700
Device Length (µm)	L	14000
O_2_ Diffusivity in O_2_ Permeable Membrane (µm^2^/s)	D_m_	160000
O_2_ Solubility in O_2_ Permeable Membrane (µM/mmHg)	K_m_	17.959
O_2_ Permeable Membrane Thickness (µm)	T	100
Hill Coefficient [11]	N	2.7
Partial Pressure at 50% Saturation [11] (mmHg)	P_50_	27
Minimum ATP Release Rate (µM/s)	R_min_	0
Maximum ATP Release Rate (µM/s)	R_max_	14
ATP Release Time (s)	t_d_	0
ATP Diffusivity in Plasma [16] (µm^2^/s)	D_ATP_	475
ATP/Luciferin Reaction Rate [8] (s^−1^)	k_t_	0.1
Quantum Efficiency [8]	α	0.88
Shutter Speed (s)	t_s_	10
Pixel Surface Area* (µm^2^)	A_pixel_	166.41
Plasma Attenuation Coefficient (µm^−1^)	κ_p_	0.1
Oxyhemoglobin Attenuation Coefficient** (µm^−1^)	κ_O2Hb_	0.040176
Hemoglobin Attenuation Coefficient** (µm^−1^)	κ_Hb_	0.066261
Camera Efficiency*	η	0.7
Full Well Capacity* (electrons)	FWC	22000

Uits are given in the first column. One asterisk (*) indicates parameters taken from the specification of Qimaging's Rolera XR camera.

Two asterisks (**) indicates parameters calculated from tabulated molar extinction coefficients for hemoglobin in water at 560 nm; these values were compiled by Scott Prahl (prahl@ece.ogi.ed).

## Results

**Figure 2:**
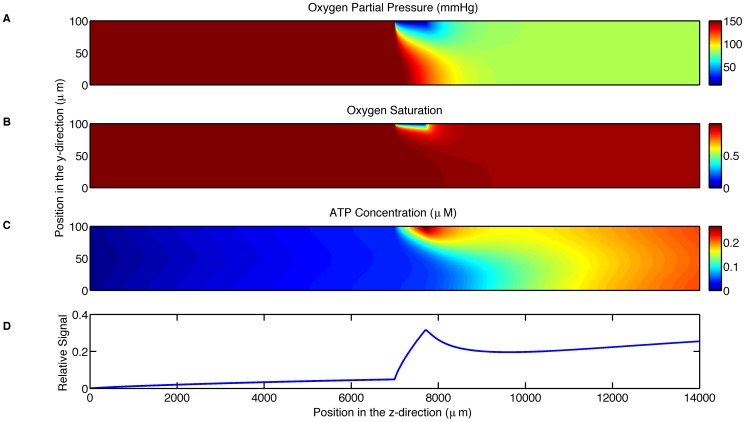
Shows the simulation results for the parameters in Table 1. A. displays the oxygen partial pressure (mmHg) as a function of position (µm) throughout the microfluidic device. B. displays hemoglobin oxygen saturation as a function of position (µm) throughout the microfluidic device. C. displays the ATP concentration (µM) as a function of position (µm) throughout the microfluidic device. D. displays the relative output signal as a function of longitudinal position.

The computational model simulated experiments with the parameters specified in Table 1. The parameters that are varied during the simulations are summarized in Table 2. The first simulation was used to test the feasibility of the experimental setup. This simulation shows that the PO_2_ decreases from 150 mmHg to below 57 mmHg in the vicinity of the oxygen permeable membrane and to 95 mmHg across the entire width of the channel 3 mm downstream from the end of the membrane (Fig. 2A). This simulation also shows that the SO_2_ decreases from 99% to below 65% in the immediate vicinity of the oxygen permeable membrane (Fig. 2B) but rapidly rises back up to 97% within 1.9 mm downstream. It should be noted that this erythrocyte resaturation occurs due to the diffusion of oxygen regions further from the membrane that were initially less affected by low O_2_ at the membrane surface. The ATP concentration, accounting for both the release from erythrocytes and degradation by luciferin/luciferase, is shown to reach a peak value of 0.2 µM in the vicinity of the oxygen permeable membrane with the concentration decreasing immediately after the end of the membrane (Fig. 2C). The relative output signal shown in Fig. 2D is the accumulated light produced by the luciferin/luciferase reaction as measured by the camera; this measurement is normalized by the maximum signal measurable by the camera for the parameter settings given in Table 1. The relative output signal begins a rapid increase from 0.032 at the position of the beginning of the membrane and reaches a maximum value of 0.191 at the end of the membrane.

**Table 2:** Model parameters varied in the simulations with the range of parameter values used in each simulation (simulation number).

Parameter	Range	Simulation Number
Device Height (µm)	25–400	2
Membrane Permeability (M µm^2^/(mmHg s))	0.06–3.84	7
Flow Rate (µL/min)	3.30–18.24	4
ATP Release Time (s)	0.00–4.00	3,4
Maximum ATP Release Rate (µM/s)	10.0–25.0	5
Minimum ATP Release Rate (µM/s)	0.0–1.5	5
ATP/luciferin Reaction Rate (s^−1^)	0.0–1.0	6

**Figure 3:**
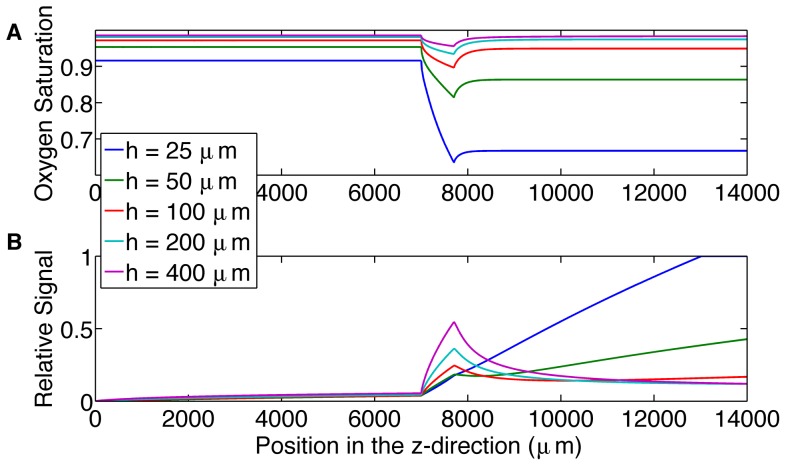
Shows the effect of increasing channel height. A. displays the mean hemoglobin oxygen saturation across the channel as a function of position (µm) along the microfluidic device for channel heights of 25, 50, 100, 200 and 400 µm. B. displays the relative output signal as a function of position (µm) for channel heights of 25, 50, 100, 200 and 400 µm.

The second simulation was used to analyze the choice of channel height and its effect on the resulting output signal (Fig. 3). All the channel heights show a rapid increase at the beginning of the oxygen-permeable membrane, this corresponds to ATP release being turned on due to the rapid desaturation of the erythrocytes. However, the 25 and 50 µm channels do not show a clear turn off of ATP release; this is because the O_2_ permeable membrane reduces O_2_ levels across the entire channel and hence the erythrocytes do not resaturate downstream of the membrane (Fig. 3A).

**Figure 4:**
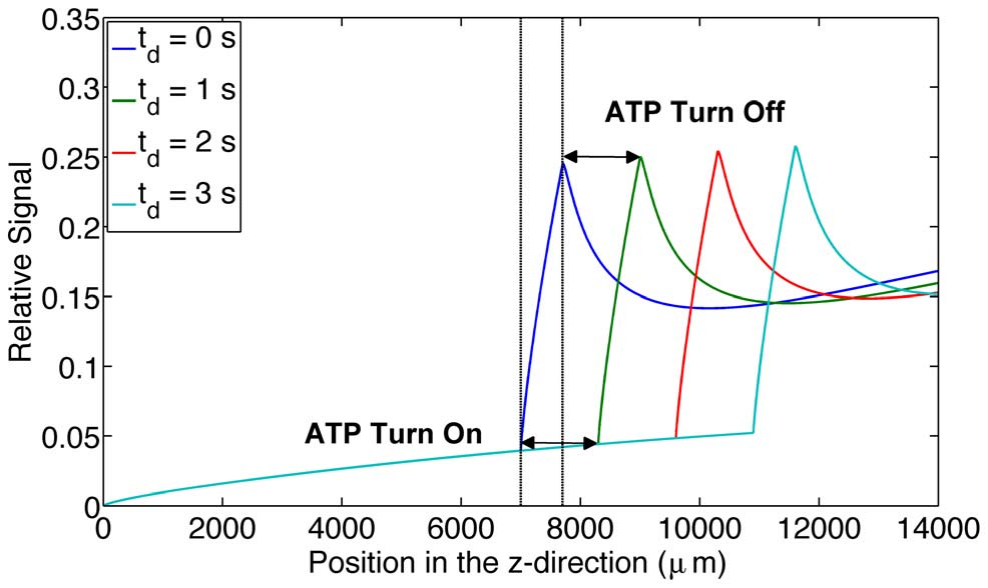
Shows the effect of increasing ATP release times. The figure displays the relative output signal for increasing ATP release times (0 s, 1 s, 2 s and 3 s) as a function of longitudinal position (µm) throughout the microfluidic device. ATP release turn on and turn off time is indicated on the graph for the 1 s delay time.

The third simulation was used to analyze the change in output signal when varying the ATP release time. Increasing release times resulted in a downstream shift in the output signal peak by 1304 µm per second of ATP release time. It should be noted that for this case the mean velocity in the channel is 1263 µm/s (Fig. 4).

**Figure 5:**
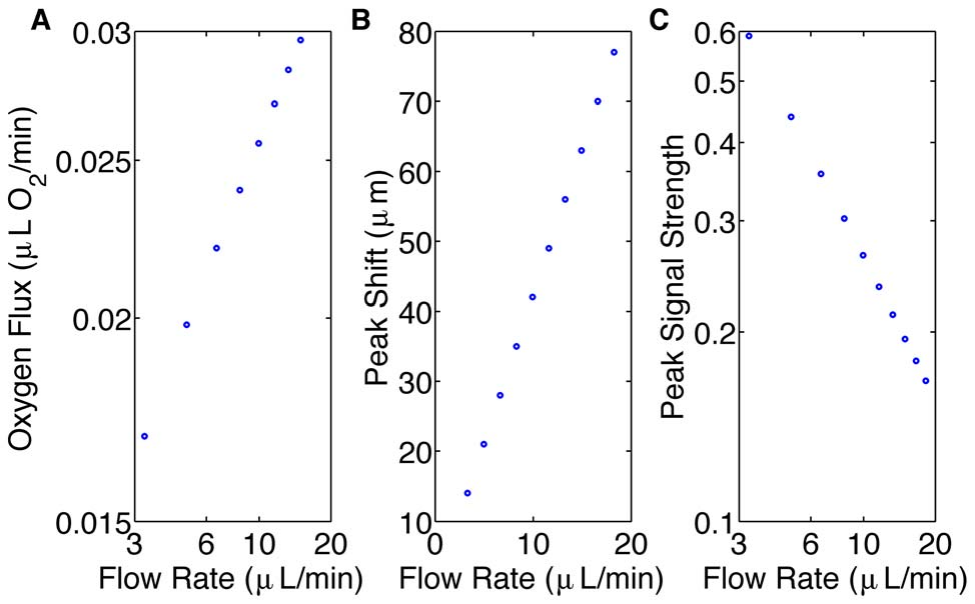
Shows the effects of flow rate on the system. A. displays the oxygen flux (µL O_2_/min) across the oxygen permeable membrane as a function of flow rate (µL/min) using a log-log scale. The data lie on a straight line with slope of 0.37. B. displays the local maximum shift (µm) caused by a 25 ms delay in ATP release as a function of flow rate (µL/min). The data lie on a straight line with slope 4.2. C. displays the magnitude of the local maximum of the output signal as a function of flow rate (µL/min) using a log-log scale. The data lie on a straight line with slope of −0.75.

The fourth simulation was used to analyze the effect of flow rate on the maximum signal strength and spatial resolution (Fig. 5). Increased flow rate resulted in an increased flux in oxygen across the oxygen permeable membrane (Fig 5A). Increased flow rate resulted in increased spatial resolution (Fig. 5B); however, it also resulted in decreased signal strength (Fig. 5C). For a 5.5 fold increase in flow rate, signal strength decreases by a factor of 3.6. For the 25-millisecond ATP release time, the peak signal shifts linearly with flow rate.

**Figure 6:**
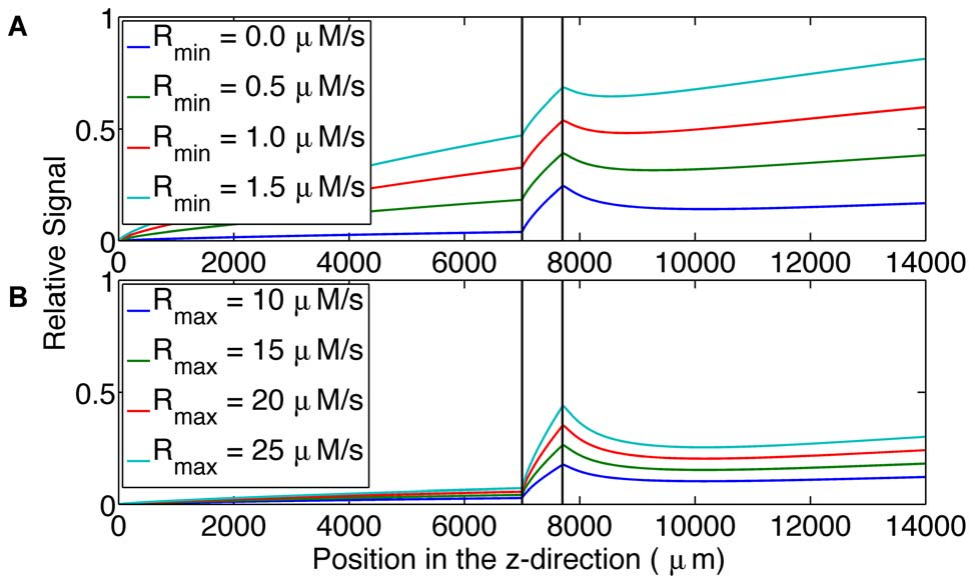
Shows the effect of ATP release rate parameters on output signal. A. displays the relative output signal for increasing minimum ATP release rates (0.0 µM/s, 0.5 µM/s, 1.0 µM/s, 1.5 µM/s) as a function of longitudinal position (µm) in the microfluidic device. B. displays the relative output signal for increasing maximum ATP release rates (10 µM/s, 15 µM/s, 20 µM/s, 25 µM/s) as a function of longitudinal position (µm) in the microfluidic device. The system is more sensitive to changes in minimum ATP release rate.

The fifth simulation considered two parameters that control the ATP release module. The first parameter determines the release rate when the erythrocyte is fully saturated; this is the minimum ATP release rate, R_min_. The second parameter determines the release rate when the erythrocyte is fully desaturated; this is the maximum ATP release rate, R_max_. The results of increasing R_min_ and R_max_ are shown in Figs 6A and 6B, respectively. Changing the minimum and maximum ATP release rates resulted in a change in the shape of the output signal.

**Figure 7:**
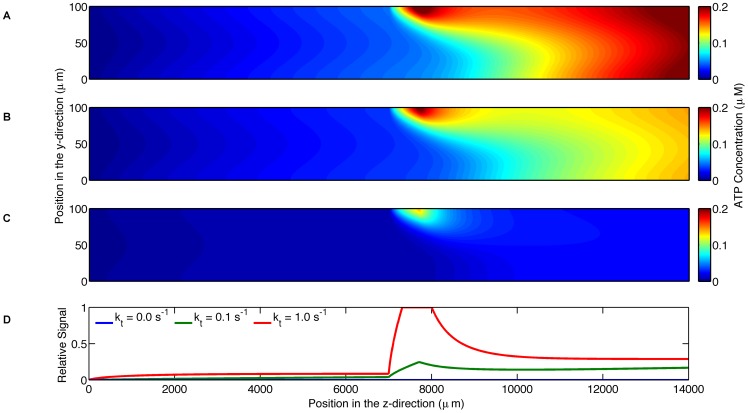
Shows the effect of ATP degradation on output signal. A. displays ATP concentration (µM) as a function of position (µm) throughout the microfluidic device where the blood does not contain the luciferin/luciferase solution. B. displays ATP concentration (µM) as a function of position (µm) throughout the microfluidic device where the blood contains the luciferin/luciferase solution with a degradation rate of 0.1 s^−1^. C. displays ATP concentration (µM) as a function of position (µm) throughout the microfluidic device where the blood contains the luciferin/luciferase solution with a degradation rate of 1 s^−1^. D. shows output signal as a function of longitudinal position along the microfluidic device for the two degradation rates. The higher degradation rate results in a larger output signal.

The sixth simulation was used to investigate the effect of the rate of the luciferin/luciferase reaction on the concentration of ATP in the channel; three degradation rates (0, 0.1, 1.0 s^−1^) were simulated. Although the case with zero degradation does not result in an output signal that can be measured by the camera, this simulation shows the ATP concentration due to the release from erythrocytes (Fig. 7A). As the degradation rate increases, ATP concentration in the channel decreases. With increasing degradation rate, the amount of light produced increases and hence the signal strength as measured by the camera also increases. This results in the apparent paradox that the highest signal strength occurs with the lowest ATP concentration in the channel. The total concentration of ATP in the channel for the zero degradation case is a factor of 1.3 and 4.9 larger than the total concentration for the degradation rates of 0.1 s^−1^ (Fig. 7B) and 1.0 s^−1^ (Fig. 7C), respectively. The maximum output signal is 5.2 fold larger with a degradation rate of 1.0 s^−1^ compared to 0.1 s^−1^ (Fig. 7D).

**Figure 8:**
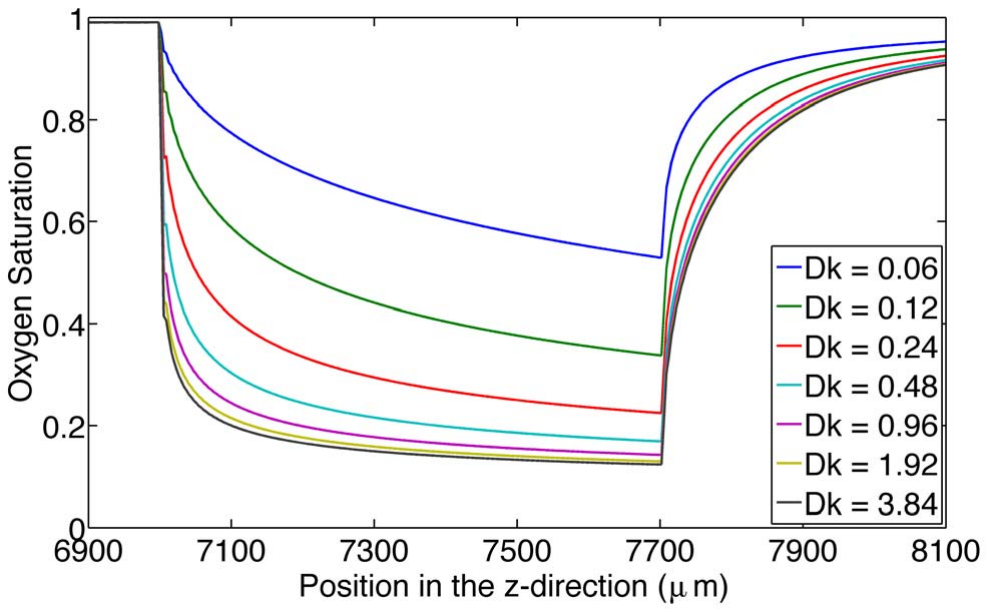
Displays the effect of the permeability of the oxygen permeable membrane on oxygen saturation. This figure shows hemoglobin oxygen saturation near the oxygen permeable membrane as a function of time (s) for increasing permeability of the oxygen permeable membrane in units M.µm^2^/(mmHg.s).

The seventh simulation was used to analyze the effect of the permeability of the oxygen permeable membrane on SO_2_ (Fig. 8). The membrane with the highest permeability (3.84 (M µm^2^)/(mmHg s)) had the ability to cause a maximum decrease from 99% to 12% whereas the membrane with the lowest permeability (0.06 (M µm^2^/(mmHg s)) was able to cause a maximum decrease from 99% to 53%.

## Discussion

The first simulation predicts that this design of a microfluidic device will be able to cause a sufficient drop in oxygen partial pressure across the width of the channel (Fig. 2A). This finding also verifies that the design of the device is effective for the purpose of causing a decrease in oxygen saturation. From this simulation, it is evident that a similar experimental procedure to that of the study of shear-dependent ATP release [3] can be repeated with this microfluidic device to study the oxygen-dependent release of ATP.

The first simulation also confirms that a digital camera will be able to measure a signal given the small concentration of ATP released from red blood cells using an exposure time of 10 seconds. The advantage of using a digital camera over a photomultiplier tube (a common method of acquiring low intensity light) is the ability to acquire all of the spatial light intensity data simultaneously at a high spatial resolution. The resulting image in combination with the simulation can be used to estimate the ATP concentration prior to degradation by the luciferin and luciferase reaction.

From the results of simulation 2 (Fig. 3), larger channel heights cause a peak in the output signal, allowing us to determine the location where ATP release turns off as the erythrocytes resaturate with oxygen from deeper in the channel.

Varying ATP release times and analyzing the corresponding output signals shows a visible change in the output signal curve. Increasing the ATP release time results in a downstream shift in the signal peak. This implies that ATP release time is measurable with this setup provided that the system remains in steady state. This allows us to use our system of high spatial resolution to obtain high temporal resolution. Measuring the distance between the beginning of the oxygen permeable membrane and the start of the rapid increase in signal gives us information about the amount of time it take for ATP release to turn on. Measuring the distance between the end of the oxygen permeable membrane and the peak in signal gives us information about the time it takes ATP release to turn off. In our model these two times are modeled as being the same, however, a recent study modeling the dynamics of the signal transduction pathway for ATP release suggests they may be different [13].

Varying flow rate and analyzing the corresponding change in the peak signal position shows that the system's resolution increases as flow rate increases, allowing us to control temporal resolution. However, the signal strength decreases with increasing flow rate. This finding demonstrates that there is a compromise between our system's resolution and the amount of measurable signal. Additionally, oxygen flux across the oxygen permeable membrane increases with increasing flow rate; this follows a power law with a power of 0.37. This finding is consistent with mass transport theory, which results in a power of 1/3 for high shear Peclet number [14]. The small discrepancy in the power may be due to the inhomogeneous distribution of oxygen throughout the oxygen permeable membrane and because the shear Peclet number for our simulations may not be sufficiently large. The shear Peclet number for oxygen transport in our simulations range from 108.78 and 601.72. Despite the increase in O_2_ flux with increasing flow rate, the signal strength decreases due to the decrease in erythrocyte transit time from leading to trailing edge of the membrane. As transit time decreases there is less time for erythrocytes to release ATP.

The flow rates used in this study (3.30–18.24 µL/min) are much smaller than the flow rate used in the study by Wan et al. (50 µL/min) [3], implying that the shear-dependent ATP release can be neglected. Varying the exposure time is another parameter that can be controlled to increase signal strength.

Another important finding from this study is that to calculate ATP release time, we need to know the velocity of the blood, which is variable across the device. Deciding which velocity to use is necessary to calculate the correct release time. This time can be calculated using our model.

ATP release rate and ATP concentration can be determined from the simulation, providing useful information about the system that would be difficult to measure directly. Comparing the simulations with and without the degradation due to the luciferin and luciferase reaction (Fig. 7), it is shown that the concentrations of ATP in the system differ by over a factor of two. This finding demonstrates that in order to determine the concentration of ATP released by the erythrocytes in a dynamic system, the calculations must account for the degradation due to the reaction. The light intensity profile measured by the camera does not directly reflect the ATP concentration produced by the oxygen dependent release; this is due to the ATP degradation by the luciferin and luciferase reaction in a flowing system. Fig. 7 demonstrates that different degradation rates can have a substantial effect on the estimated ATP concentrations indicating that one must carefully determine what the ATP degradation rate is for the experimental system used. Relying solely on the measured light intensity signal may results in misinterpretation of the ATP release dynamics.

From the results of the sixth simulation (Fig. 8), it is demonstrated that with increasing O_2_ diffusivity of the O_2_ permeable membrane, the drop in saturation becomes independent of the diffusivity.

Varying the minimum and maximum ATP release rates independently and analyzing the resulting output signal shows a measurable signal difference between the different parameters of the ATP release module. This finding supports the objective of being able to study the oxygen-dependent release of ATP because the model allows for the differentiation of individual components of the ATP release module. Further, the model can be used with the experimental procedure to adjust the three parameters (minimum and maximum release rate and release time) in the model so they match the experimental results.

A recent study suggests that ATP release may be proportional to the rate of oxygen desaturation rather than the magnitude of the saturation [13]. By varying the oxygen permeability of the oxygen permeable membrane, the model shows that the rate of desaturation may be controlled. By varying the length of the membrane, we can control the magnitude of desaturation; this will allow for the verification of this theory (see Fig. 8).

As we gain a better understanding of the dynamics of the signaling pathway for ATP release, these can be incorporated into our simulation. Ultimately, model parameters could be varied to yield the best fit with experimental measurements. Improving the model of ATP release will allow it to be used for modeling local oxygen regulation in the microvasculature *in vivo* and for the design and interpretation of *in vivo* experiments.

The results of such experiments can then be compared to the ATP release magnitude and dynamics that occur in cardiovascular disease. Finding differences between the ATP release magnitude and dynamics will allow the device combined with the model to be used to screen blood from patients with cardiovascular disease to determine whether cardiovascular disease is present.

In conclusion, a microfluidic device can be designed to produce a rapid decrease in the oxygen saturation of erythrocytes across the width of the channel resulting in a measurable ATP signal that can be analyzed for the dynamics of oxygen saturation-dependent ATP release. In addition, this computational model is an effective tool to optimize the microfluidic experiment to be able to determine the time course of ATP release from erythrocytes. Further, it can be used to determine other unknown parameters such as the minimum and maximum ATP release rate. The model may also be used in the analysis of experimental results that may be difficult to interpret, such as determining the concentration of ATP in the system prior to the degradation from the luciferin and luciferase reaction and the conversion of the spatial displacement of ATP release into ATP release time. The model can also be modified based on experimental results to further develop the model leading to a better understanding of the ATP release pathway. Further, this model is testable; experimental measurements of oxygen saturations [15] and ATP concentrations [7] can be made to justify the results of the model. In future studies, the model may be also used for the analysis of systems *in vivo* where parameter may often be very difficult to measure.
